# Prognostic Significance of Mean Platelet Volume on Local Advanced Non-Small Cell Lung Cancer Managed with Chemoradiotherapy

**DOI:** 10.1038/s41598-019-40589-4

**Published:** 2019-03-08

**Authors:** Abdullah Sakin, Saban Secmeler, Serdar Arici, Caglayan Geredeli, Nurgul Yasar, Cumhur Demir, Ferdi Aksaray, Sener Cihan

**Affiliations:** 1 Department of Medical Oncology, Yuzuncu Yil University Medical School, 65030 Van, Turkey; 2 Department of Medical Oncology, University of Health Sciences, Okmeydani Training and Research Hospital, 34384 Istanbul, Turkey; 3 Department of Radiation Oncology, University of Health Sciences, Okmeydani Training and Research Hospital, 34384 Istanbul, Turkey

## Abstract

Mean platelet volume (MPV), the most commonly used measure of platelet size, and is altered in patients with malignancies. The aim of this study was to investigate the effect of MPV on overall survival (OS) of patients with locally advanced (Stage IIIA/B) inoperable non-small cell lung cancer (NSCLC). This retrospective study included patients who received concomitant chemoradiotherapy (CCRT) with cisplatin + etoposide regimen due to locally advanced stage IIIA/B NSCLC. The study included a total of 115 cases, consisting of 110 (95.7%) male and 5 (4.2%) female patients. The mean age of the patients was 61.3 ± 10.4 (22–82) years. ROC curve generated by MPV for OS yielded an AUC of 0.746 (95% CI 0.659–0.833), (p < 0.001). MPV was detected as >9 fL with a sensitivity of 74.4% and a specificity of 72.0%. In patients with stage IIIA, median OS was 45.0 months (95% CI 17.3–74.1) and 21 months (95% CI 10.6–31.3) in groups with MPV > 9.0 fL and ≤9.0 fL, respectively (p = 0.013). In patients with stage IIIB, median OS was 44.0 months (95% CI 13.8–60.6) and 16 months (95% CI 9.5–22.4) in groups with MPV > 9.0 fL and ≤9.0 fL, respectively (p = 0.036). ECOG performance score, total platelet count, and MPV were found as the most significant independent factors affecting survival (p < 0.001, p = 0.008, and, p = 0.034, respectively). In this study, we showed that decreased pre-treatment MPV was an independent risk factor for survival in NSCLC patients who were administered CCRT. As part of routine complete blood count panel, MPV may represent one of the easiest measuring tools as an independent prognostic marker for survival in locally advanced NSCLC.

## Introduction

Lung cancer is the most common cancer in the world and is the number one reason for cancer-related mortality in women and men^1^. Non-small cell lung cancer (NSCLC) accounts for 85% of all lung cancer cases. Stage III NSCLC contains a heterogeneous group of patients, where the choice of treatment remains controversial^2^. Despite radical surgery, radiotherapy (RT), chemotherapy, and recent modalities such as targeted therapy or immunotherapy, the prognosis is still poor with a 5-year survival rate below 15%^3^. Therefore, investigation of new biomarkers is needed to develop treatment strategies.

Platelet volume is determined during both megakaryopoiesis and thrombopoiesis. Megakaryocytic maturation, platelet production, and platelet size can be modulated by cytokines such as interleukin-6 (IL-6), granulocyte colony-stimulating factor (G-CSF) and macrophage colony-stimulating factor (M-CSF)^4^, Mean platelet volume (MPV), the most commonly used measure of platelet size, is a marker of platelet activation and is associated with various inflammatory conditions^5^. Large platelets could be more easily stimulated for release of chemical mediators; therefore, they are considered more reactive than small platelets^6^. The studies performed so far reported thrombocytosis as an unfavorable prognostic factor for overall survival (OS) in NSCLC^7,8^. In addition, an earlier study reported that lower MPV was significantly associated with decreased OS in operated NSCLC patients^9^.

This study aimed to investigate the effect of MPV on OS in patients who received concurrent chemoradiotherapy with cisplatin + etoposide regimen due to locally advanced stage (IIIA/B) NSCLC.

## Results

The study included 115 cases, consisting of 110 (95.7%) male and 5 (4.2%) female patients. The mean age of the patients was 61.3 ± 10.4 (range: 22–82) years. Forty (34.8%) patients were ≥65 years old. Most of patients (93.9%, n = 108) had history of smoking, and among them, 63 (54.8%) patients had smoked >40 pack-years. ECOG PS score was found as 2 in 31 (27%) patients. Distribution of comorbidities showed 37 patients (32.2%) with hypertension (HT) and six patients (5.2%) with diabetes (DM). The mean body mass index (BMI) was 24.4 ± 4.9 kg/m^2^ (14.1–40.0). Analysis of tumor histology showed 23 cases (20%) with undetermined histologic subtype, 63 cases (54.8%) with squamous cell carcinoma, and the remaining 29 cases (25.4%) with adenocarcinoma. Stage IIIA and IIIB patients constituted 36.5% (n = 42) and 63.5% (n = 73) of the study population, respectively. Seventy-four patients (64.3%) had died during the median 16.2 months’ follow-up (Table 1).

**Table 1:** Patients’ characteristics by MPV groups.

		All patients (n = 115)		MPV ≤ 9 (n = 65)		MPV > 9 (n = 50)		
		n	%	n	%	n	%	p
Gender	Female	5	4.3	4	6.2	1	2	0.258
	Male	110	95.7	61	93.8	49	98	
Age (years)	Mean ± SD (min-max)	61.30 ± 10.40 (22–82)		61.53 ± 10.25 (40–82)		61.0 ± 10.67 (22–80)		0.784
	<65	75	65.2	43	66.2	32	64	0.810
	≥ 65	40	34.8	22	33.8	18	36	
Smoking status	No	7	6.1	6	9.2	1	2	0.136
	Yes	108	93.9	59	90.8	49	98	
	≤40 (pack-years)	52	45.2	30	46.2	22	44	0.818
	>40 (pack-years)	63	54.8	35	53.8	28	56	
ECOG PS status	0–1	84	73	43	66.2	41	82	0.058
	2	31	27	22	33.8	9	18	
HT	No	78	67.8	46	70.8	32	64	0.441
	Yes	37	32.2	19	29.2	18	36	
DM	No	109	94.8	62	95.4	47	94	0.741
	Yes	6	5.2	3	4.6	3	6	
BMI (kg/m^2^)	Mean ± SD (min-max)	24.48 ± 4.91 (14.19–40.06)		22.98 ± 4.37 (14.19–40.06)		26.12 ± 5.0 (16.44–37.64)		**0.002**
Tumor histology	Undetermined subtype	23	20	13	20	10	20	0.984
	Squamous cell carcinoma	63	54.8	36	55.4	27	54	
	Adenocarcinoma	29	25.2	16	24.6	13	26	
Tumor stage	IIIA	42	36.5	18	27.7	24	48	**0**.**025**
	IIIB	73	63.5	47	72.3	26	52	
Final status	Alive	41	35.7	11	16.9	30	60	**<0.001**
	Dead	74	64.3	54	83.1	20	40	

**Abbreviations: BMI**, body mass index; **ECOG PS**, Eastern Cooperative Oncology Group Performance Status; **DM**, diabetes mellitus; **HT**, hypertension; **Max**, maximum**; Min**, minimum**; MPV**, mean platelet volume.

No statistically significant difference was found between MPV groups in terms of mean FPG, creatinine, AST, ALT, GGT, LDH, sodium, potassium, calcium, albumin, WBC, Hb, RDW, TNC, TLC, and TMC. Mean TPC and PDW were significantly higher in MPV ≤ 9 fL group than those in MPV > 9 fL group (p = 0.005 and p = 0.027, respectively), (Table 2).

**Table 2:** Laboratory values by MPV groups.

		Total		MPV ≤ 9 (n = 65)		MPV > 9 (n = 50)		
		N	Mean ± SD (min-max)	N	Mean ± SD (min-max)	N	Mean ± SD (min-max)	p
FPG	(mg/dL)	109	110.3 ± 48.8 (10–292)	60	107.7 ± 37.8 (64–287)	49	113.6 ± 50.5 (10–292)	0.482
Creatinine	(mg/dL)	107	0.9 ± 0.2 (0.4–1.8)	60	0.9 ± 0.2 (0.5–1.7)	47	0.9 ± 0.2 (0.4–1.8)	0.601
AST	(U/L)	101	18.0 ± 7.3 (7–53)	55	18.1 ± 6.9 (7–41)	46	17.8 ± 7.8 (8–53)	0.851
ALT	(U/L)	101	17.9 ± 13.4 (1–100)	55	16.2 ± 9.7 (2–49)	46	20.0 ± 16.7 (1–100)	0.159
ALP	(U/L)	80	108.6 ± 51.1 (45–321)	47	107.7 ± 46.9 (48–307)	33	109.9 ± 57.2 (45–321)	0.852
GGT	(U/L)	72	56.3 ± 73.1 (9–438)	43	57.9 ± 65.8 (13–333)	29	53.8 ± 83.9 (9–438)	0.817
LDH	(U/L)	73	257.6 ± 274.6 (117–2358)	47	291.5 ± 334.8 (117–2358)	26	196.5 ± 69.9 (127–458)	0.158
Sodium	(mmol/L)	59	138.4 ± 3.7 (125–148)	32	137.8 ± 3.8 (125–142)	27	139.1 ± 3.6 (128–148)	0.168
Potassium	(mmol/L)	59	4.6 ± 0.5 (3.6–5.8)	29	4.6 ± 0.5 (3.7–5.7)	30	4.5 ± 0.4 (3.6–5.8)	0.641
Calcium	(mg/dL)	55	9.3 ± 0.5 (7.7–10.6)	30	9.3 ± 0.5 (8.2–10.5)	25	9.4 ± 0.5 (7.7–10.6)	0.352
Albumin	(g/dL)	41	3.7 ± 0.6 (2.4–4.6)	21	3.6 ± 0.5 (2.63–4.6)	20	3.8 ± 0.6 (2.0–4.6)	0.399
WBC	(10^9^/U)	115	9.3 ± 2.9 (4.9–18.0)	65	9.4 ± 2.9 (4.9–18.0)	50	9.0 ± 3.0 (4.9–16.2)	0.530
Hb	(g/dL)	115	12.5 ± 1.9 (7.96–17.10)	65	12.3 ± 1.8 (7.96–10.97)	50	12.8 ± 2.0 (8–17.1)	0.214
TPC	(10^9^/U)	115	345.4 ± 121.4 (76.1–696.0)	65	373.2 ± 120.1 (211–696)	50	309.1 ± 114.3 (76.1–647.0)	**0.005**
PDW	(%)	110	16.6 ± 9.6 (8.2–63.3)	61	18.4 ± 10.6 (8.2–54.2)	49	14.3 ± 7.6 (10.0–63.3)	**0.027**
MPV	(fL)	115	8.8 ± 1.33 (5.8–13.2)	65	7.9 ± 0.8 (5.86–9.0)	50	9.9 ± 0.8 (9.1–13.2)	**<0.001**
RDW	(%)	97	14.9 ± 2.3 (11.0–25.0)	47	15.4 ± 2.7 (11.9–25.0)	50	14.5 ± 1.9 (11.0–21.0)	0.057
TNC	(10^9^/U)	115	6.4 ± 2.4 (2.1–14.4)	65	6.7 ± 2.4 (3.02–14.40)	50	6.1 ± 2.4 (2.1–10.9)	0.19
TLC	(10^9^/U)	115	1.9 ± 0.8 (0.3–4.5)	65	1.7 ± 0.8 (0.34–4.05)	50	2.0 ± 0.8 (0.3–4.5)	0.086
TMC	(10^9^/U)	114	0.7 ± 0.3 (0.1–2.4)	64	0.7 ± 0.3 (0.1–1.34)	50	0.7 ± 0.4 (0.1–2.4)	0.933

**Abbreviations: ALT**, alanine aminotransferase; **AST**, aspartate aminotransferase; **fL**, femtoliter; **FPG**, fasting plasma glucose; **Hb**, hemoglobin; **GGT**, gamma-glutamyl transferase; **LDH**, lactate dehydrogenase; **mg/dL**, milligram/decilNiter; **MPV**, mean platelet volume; **N**, number of patients; **PDW**, platelet distribution width; **RBC**, red blood cell**; RDW**, red blood cell distribution width; **U/L**, Units per liter; **TLC**, total lymphocyte count; **TMC**, total monocyte count; **TNC**, total neutrophil count; **TPC**, total platelet count, **WBC**, white blood cell.

**Figure 1:**
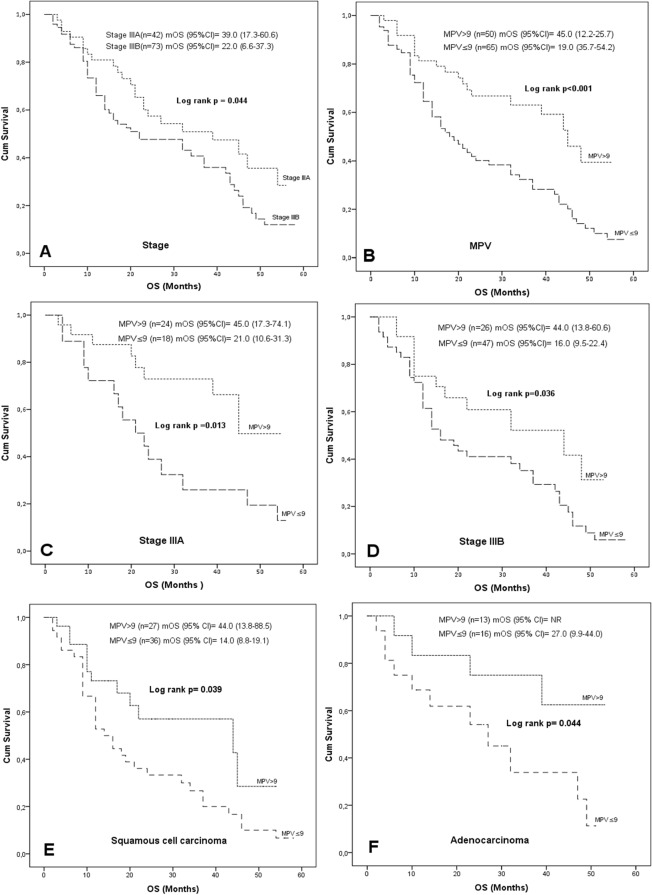
(**A)** Overall survival by clinical stages, (**B)** Overall survival by MPV groups in all patients (**C)** Overall survival by MPV groups in stage IIIA, (**D)** Overall survival by MPV groups in stages IIIB, (**E)** Overall survival by MPV groups in squamous cell carcinoma,(**F)** Overall survival by MPV groups in adenocarcinoma. **Abbreviations**: **CI**, *confidence interval*; **MPV**: mean platelet volume, **mOS**: median overall survival, **N**, number of patients; **OS**: overall survival.

In all patients, median OS was 45.0 months (95% CI 35.7–54.2) and 19.0 months (95% CI 12.2–25.7) in groups with MPV > 9.0 fL and ≤9.0 fL, respectively (log-rank p < 0.001). Analysis by stages showed median OS as 39.0 months (95% CI 17.3–60.6) and 22.0 months (95% CI 6.6–37.3) in patients with stage IIIA and stage IIIB, respectively (log-rank p = 0.044). In patients with stage IIIA, median OS was found as 45.0 months (95% CI 17.3–74.1) and 21.0 months (95% CI 10.6–31.3) in groups with MPV > 9.0 fL and ≤9.0 fL, respectively (log-rank p = 0.013). In patients with stage IIIB, median OS was detected as 44.0 months (95% CI 13.8–60.6) and 16.0 months (95% CI 9.5–22.4) in groups with MPV > 9.0 fL and ≤9.0 fL, respectively (log-rank p = 0.036). With regard to tumor histology: In patients with squamous cell carcinoma, median OS was detected as 44.0 months (95% CI 13.8–88.5) and 14.0 months (95% CI 8.8–19.1) in groups with MPV > 9.0 fL and ≤9.0 fL, respectively(log-rank p = 0.039). In patients with adenocarcinoma median OS was not reached and 27.0 months (95% CI 9.9–44.0) in groups with MPV > 9.0 fL and ≤9.0 fL, respectively (log-rank p = 0.044) (Fig. 1).

In univariate analysis, ECOG PS score, HT, BMI, stage, Hb, TPC, and MPV were found as the most significant independent factors affecting survival (p < 0.001, p = 0.040, p = 0.022, p = 0.045, p = 0.023, p = 0.005, and p = 0.001, respectively). On the other hand, ECOG PS score, TPC, and MPV were the most significant independent factors affecting survival in multivariate analysis (p < 0.001, p = 0.008, and, p = 0.034, respectively), (Table 3).

**Table 3:** Predictors of the survival.

		Univariate analysis for OS			Multivariate analysis for OS		
		HR	95% CI	p	HR	95%	p
Gender	Male vs. female	1.176	0.427–3.238	0.754			
Age at diagnosis	Years	1.012	0.988–1.036	0.320			
Smoking status	Current vs. never	0.778	0.336–1.799	0.557			
	>40 vs.≤40 pack-years	1.117	0.704–1.769	0.639			
ECOG PS	2 vs. 0–1	5.132	3.131–8.409	**<0.001**	4.428	2.373–8.264	**<0.001**
HT	No vs. yes	0.547	0.329–0.908	**0.040**			
DM	No vs. yes	0.629	0.197–2.002	0.433			
BMI	kg/m^2^	0.932	0.877–0.990	**0.022**			
Tumor histology	Undetermined subtype	1.000		**0.113**			
	Squamous carcinoma	1.613	0.895–2.905	0.112			
	Adenocarcinoma	0.943	0.460–1.933	0.873			
Stage	IIIB vs. IIIA	1.645	1.001–2.702	**0.045**			
FPG	(mg/dL)	0.997	0.990–1.002	0.259			
Creatinine	(mg/dL)	0.821	0.313–2.149	0.687			
AST	(U/L)	1.008	0.972–1.045	0.662			
ALT	(U/L)	1.004	0.984–1.025	0.678			
ALP	(U/L)	1.002	0.995–1.008	0.546			
GGT	(U/L)	1.002	0.999–1.006	0.187			
LDH	(U/L)	1.000	0.999–1.001	0.227			
Sodium	(mmol/L)	0.969	0.894–1.051	0.449			
Potassium	(mmol/L)	1.147	0.565–2.330	0.706			
Calcium	(mg/dL)	0.955	0.515–1.769	0.883			
Albumin	(g/dL)	0.649	0.352–1.193	0.164			
WBC	(10^9^/U)	1.055	0.978–1.137	0.164			
Hb	(g/dL)	0.868	0.767–0.981	**0.023**			
TPC	(10^3^/U)	1.003	1.000–1.005	**0.005**	1002	1000–1005	**0,041**
PDW	(%)	1.021	0.999–1.044	0.053			
MPV	(fL)	0.767	0.646–0.910	**0.001**	0.807	0.662–984	**0.034**
RDW	(%)	1.081	0.984–1.125	0.103			
TNC	(10^9^/U)	1.071	0.980–1.171	0.129			
TLC	(10^9^/U)	0.880	0.668–1.158	0.362			
TMC	(10^9^/U)	1.840	0.897–3.771	0.096			

**Abbreviations:** see Table 1 and Table 2.

## Discussion

A number of prognostic factors has been investigated to predict the clinical course in NSCLC yet many of them could be used in clinical practice^10,11^. In this study, the association of MPV to survival in non-metastatic inoperable NSCLC was evaluated, where a low MPV was determined as an unfavorable risk factor.

Recently, association of thrombocytosis to the poor prognosis has been reported in colon, stomach, endometrial, and lung cancer^12–15^. In the last decade, several studies have suggested that platelet activation represents an important biological process in metastasis and carcinogenesis^12^. Previous studies investigated prognostic effect of platelet counts in patients with NSCLC and reported that platelet counts could be used as a prognostic factor. Supporting these reports, increased platelet counts was found to be an independent risk factor in our study. Nevertheless, our study population consisted of locally advanced patients, unlike other studies^8,9,16^.

As it was reported to be associated with platelet aggregation, the release of thromboxane B2, and increased expression of platelet adhesions molecule glycoprotein IIB/IIIA, MPV is thought to reflect platelet activity^17–19^.

A study reported that microparticles from P-selectin-bearing lung cancer cells activated tissue factor carrying platelets and led to thrombus formation^20^. The tendency of larger platelets to respond stimuli renders them to be selectively consumed, causing decreased MPV of circulating platelets. Recent studies have suggested alteration of MPV levels in various diseases, including a number of cancers^9,16,21^. Several clinical trials reported increased MPV to be associated with thromboembolic diseases such as myocardial infarction^21^. A significant reduction in MPV was demonstrated in cancer patients with bone marrow metastasis compared to control group^22^. Mutlu *et al*. examined MPV levels in various types of cancer and reported significantly decreased MPV levels during thrombotic events^23^. In addition, Osada *et al*. reported that MPV was statistically higher in patients with stomach cancer compared to healthy volunteers^24^. Shen *et al*. reported optimal MPV cut-off value for survival as 4 fL with a 28.2% sensitivity and 85.2% specificity in the study they performed on patients with esophagus cancer. They detected 5-year OS as 47% in the group with high MPV compared to 28.3% in the group with low MPV^25^.

Kumagai *et al*. reported to detect preoperative low MPV as an independent poor prognostic factor in their study with 308 Japanese resected NSCLC patients, where they found the optimal cut-off value as 8.5 fL with a sensitivity of 87.2% and a specificity of 44.1%. The authors further reported 5-year OS as 93.3% in the group with MPV ≥ 8.50 fL compared to 73.6% in the group with MPV < 8.50 fL (p = 0.001)^9^. In addition, Gao *et al*. in their study of 546 operated Chinese patients, found preoperatively increased TPC and decreased MPV as unfavorable risk factor for survival. They determine the cut-off value of MPV as 11.0. Those with MPV < 11 fL and TPC ≥ 300 × 10^9^ had a 5-year OS of 27.9%, which was significantly lower in those with MPV value above the cut-off point (p < 0.001)^26^. Inagaki *et al*. reported low MPV/TPC ratio as unfavorable risk factor for OS in the study they performed with 268 advanced stage NSCLC patients. In this study, the number of patients in stage III was only 15^16^. Interestingly, a recent study has reported that increased MPV negatively influenced progression-free survival in patients with metastatic stage EGRF mutant NSCLC adenocarcinoma^27^.

Consistent with those in the literature, our study detected declined MPV as an independent risk factor for OS. We determined cut-off value of MPV as 9.0 fL with 74.4% sensitivity and 72.0% specificity. Median OS was 45 months in patients with a MPV value of above 9.0 fL and 19 months in those with MPV ≤ 9.0 (p < 0.001). In stage IIIA stratum, these were 45 months and 21 months, respectively (p = 0.013). Similarly, median OS were 44 months and 16 months, respectively, in patients with stage IIIB NSCLC (p < 0.036).

Contrary to previous studies in the literature, our study included patients with locally advanced disease managed with CCRT^7–9,16,26,27^. Nonetheless, the limitations of current study were its retrospective design and comparably lower sample size. Further studies are warranted to elucidate the exact mechanisms how MPV affects prognosis of patients with locally advanced NSCLC.

In conclusion, we showed that low pre-treatment MPV was an independent risk factor for survival in NSCLC patients who received cisplatin plus etoposide-based CCRT. As part of routine complete blood count panel, MPV may represent one of the easiest measuring tools as an independent prognostic marker for survival in locally advanced NSCLC.

## Methods

### Study population

This retrospective study included subjects who received CRT due to locally advanced NSCLC in the department of oncology in Okmeydani Training and Research Hospital, Istanbul between years of 2010 and 2017. Cases with malignancies other than NSCLC, hematological disease, early stage or metastatic disease, infection before the treatment, or using immunosuppressive drugs or acetylsalicylic acid, or those who had incomplete data were not included to the study. Study population consisted of >18-year-old patients who were administered concomitant CRT (CCRT) with cisplatin plus etoposide regimen due to inoperable stage IIIA/B with no distant metastasis. Staging in our hospital is based on pre-treatment 18-fluorodeoxyglucose positron emission tomography/computed tomography (PET/CT), computed tomography, cranial magnetic resonance, and mediastinoscopy findings. Routine blood tests were obtained in all patients by venous sampling after 8 hours of fasting before treatment. For routine biochemical tests, tubes not containing anticoagulants were used and for hemogram we used tubes containing ethylenediamine tetraacetic acid. Complete blood counts were done using by homogram autoanalyzer (Mindray, BC6800 model, China). Biochemical tests were performed by colorimetric method in autoanalyser (Beckman Coulter Brand, AU 5800 model, USA). CCRT consists of cisplatin (50 mg/m^2^ on days 1, 8, 29, and 36) + etoposide (on days 1–5 and 29–33) regimen. In CCRT, thoracic RT was applied as a total of 60 Gy in 30 fractions of 2 Gy/day.

### Data collection

**Figure 2:**
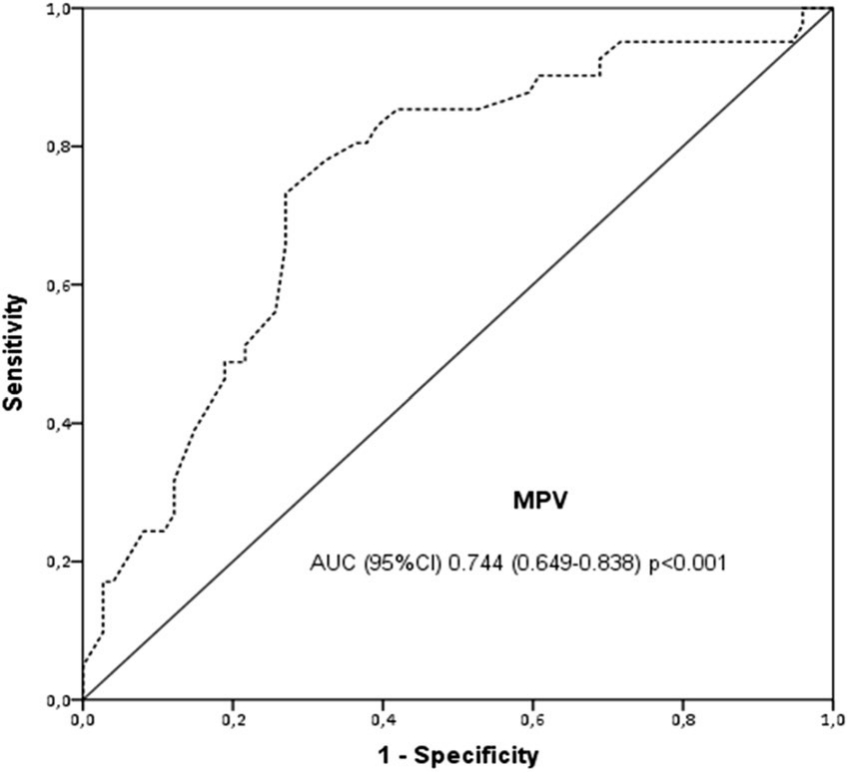
ROC curve analyses for overall survival by MPV is represented by the line with an AUC = 0.746 (95%CI 0.659–0.833, p < 0.001), where the MPV cut-off value was detected as 9.0 fL with a sensitivity of 74.4% and a specificity of 72.0%. ***Abbreviations***: **AUC**, area under the curve; **CI**, *confidence interval*; ***MPV***, *m*ean platelet volume.

Age, gender, smoking, ECOG PS, comorbidities, histological type, planned treatment, and final status of the patients were obtained from the medical records. Similarly, pre-treatment laboratory parameters including fasting plasma glucose (FPG, creatinine, aspartate aminotransferase (AST), alanine aminotransferase (ALT), alkaline phosphatase (ALP), gamma-glutamyl transferase (GGT), lactate dehydrogenase (LDH), sodium, potassium, calcium, albumin, white blood cell (WBC) count, red blood cell (RBC) count, hemoglobin (Hb), hematocrit (Hct), MPV, total platelet count (TPC), total neutrophil count (TNC), total lymphocyte count (TLC), total monocyte count (TMC) were recorded from initial assessment after diagnosis. ROC curve generated for OS by MPV yielded an AUC of 0.746 (95% CI 0.659–0.833, p < 0.001). MPV was detected as >9 fL with a sensitivity of 74.4% and a specificity of 72.0% (Fig. 2). Patients were divided into two groups as having MPV ≤ 9 fL and MPV > 9 fL. Several characteristics of cases were also group as such: the age as <65 or ≥65 years old; ECOG PS as 0–1 or 2; and smoking as non-smoker or smoker and smoking ≤40 or >40 pack-years.

### Statistical analysis

SPSS 15.0 for Windows software was used for the statistical analysis. Descriptive statistics were presented as the mean, standard deviation, minimum, and maximum values for numerical variables; and as number and percentage for categorical variables. Numerical variable between two independent groups were analyzed with student t-test in case of normal distribution and with Mann Whitney U test if else. The comparison of the rates between the groups was performed by chi-square analysis. Monte Carlo simulation was applied if conditions could not be met. Survival analyses were performed with Kaplan-Meier Analysis. Determinant factors were examined with cox regression. Backward stepwise model was used with parameters having a p-value below 0.100. Cut-off value was determined with receiver operating characteristics (ROC) curve analysis. An overall 5% alpha error level was used to infer statistical significance.

### Institutional review board statement

The study was performed in accordance with the declaration of Helsinki and was reviewed and approved by the Ethics Committee of the University of Health Sciences, Okmeydani Training and Research Hospital (26.02.2018).

### Informed consent statement

Patients were not required to give informed consent to the study because the analysis used anonymous clinical data that were obtained after each patient agreed to treatment by written consent.

## Data Availability

All relevant data are available in the supplementary materials.
